# LigA formulated in AS04 or Montanide ISA720VG induced superior immune response compared to alum, which correlated to protective efficacy in a hamster model of leptospirosis

**DOI:** 10.3389/fimmu.2022.985802

**Published:** 2022-10-10

**Authors:** Vivek P. Varma, Mohammad Kadivella, Ajay Kumar, Sridhar Kavela, Syed M. Faisal

**Affiliations:** ^1^ Laboratory of Vaccine Immunology, National Institute of Animal Biotechnology, Hyderabad, India; ^2^ Graduate Studies, Manipal Academy of Higher Education, Manipal, Karnataka, India; ^3^ Regional Centre for Biotechnology, Faridabad, India

**Keywords:** LIGA, clinical adjuvant, vaccine, AS04, Montanide ISA720VG, leptospirosis

## Abstract

Leptospirosis is a zoonotic disease of global importance. The current vaccine provides serovar-specific and short-term immunity and does not prevent bacterial shedding in infected animals. Subunit vaccines based on surface proteins have shown to induce protection in an animal model. However, these proteins were tested with non-clinical adjuvants and induced low to moderate protective efficacy. We formulated a variable region of *Leptospira* immunoglobulin-like protein A (LAV) in clinical adjuvants, AS04 and Montanide ISA720VG, and then evaluated the immune response in mice and protective efficacy in a hamster model. Our results show that animals immunized with LAV-AS04 and LAV-Montanide ISA720VG (LAV-M) induced significantly higher levels of LAV-specific antibodies than LAV-Alum. While LAV-Alum induced Th2 response with the induction of IgG1 and IL-4, AS04 and LAV-M induced a mixed Th1/Th2 response with significant levels of both IgG1/IL-4 and IgG2c/IFN-γ. Both LAV-AS04 and LAV-M induced the generation of a significantly higher number of cytotoxic T cells (CTLs). The immune response in LAV-AS04- and LAV-M-immunized animals was maintained for a long period (>180 days) with the generation of a significant level of B- and T-cell memory. The strong immune response by both vaccines correlated to enhanced recruitment and activation of innate immune cells particularly DCs at draining lymph nodes and the formation of germinal centers (GCs). Furthermore, the immune response generated in mice correlated to protective efficacy in the hamster model of leptospirosis. These results indicate that LAV-AS04 and LAV-M are promising vaccines and can be further evaluated in clinical trials.

## Introduction

Leptospirosis is a life-threatening zoonotic disease caused by a Gram-negative spirochete *Leptospira* that occurs throughout the world with the highest incidence in tropical regions. More than 1 million cases of severe leptospirosis occur each year, with case fatality rates exceeding 10% (1, 2). It has become one of the significant causes of morbidity and mortality worldwide, especially in impoverished populations. The disease spectrum caused by leptospiral infection is vast and varies from sub-clinical inapparent to severe multi-organ syndrome involving liver, kidney, and lungs either alone or in combination (3). Pulmonary hemorrhage is increasingly recognized as a significant complication in several outbreaks of leptospirosis in developing countries (4). The disease is misdiagnosed as the symptoms overlap with the common flu, dengue fever, hantavirus infection, encephalitis, viral hepatitis, malaria, and COVID-19 (5, 6). Vaccination is the most effective strategy to control the disease, but no vaccine is available for humans. The available vaccine is inactivated whole bacterin, which is used in livestock and companion animals, and in some countries, it is used in humans who are at risk (7, 8). These vaccines induce short-term and serovar-specific immunity, are associated with toxicity, and do not provide sterilizing immunity (8). Hence, there is a need to develop a safe and effective vaccine that can induce long-term cross-protection and prevent transmission to a susceptible host.

In recent years, subunit vaccines based on outer membrane/surface proteins have become an attractive alternative to the whole-cell vaccine, and several candidates have been identified that induced protection in a hamster model of the disease (9–18). Of these, *Leptospira* immunoglobulin-like protein A (LigA) is the most promising candidate and several investigators have established its protective role in the hamster model of leptospirosis (19–25). Furthermore, the C terminal or variable region of LigA (LAV), specifically domains 10–13, was shown to be sufficient to induce protection against disease in the hamster model (20, 26).

Adjuvants are key to the success of subunit vaccines and alum is most widely used in licensed vaccines for various bacterial and viral diseases (27). Although alum has been tested with different subunit antigens for *Leptospira*, it induced mainly humoral and limited Th1 response and failed to induce sterilizing immunity (10, 20, 24, 28–30). Other adjuvants like Freund’s, liposomes, xanthan gum, PLGA-microparticles, and emulsions like AddaVax and Emulsigen-D and Salmonella flagellin have also been tested with various surface proteins of *Leptospira* including LigA (8, 10, 17, 19, 21, 22, 28–32). Although these adjuvants induced significantly higher level of antibodies and T-cell response than alum, they are preclinical adjuvants that have not been approved for human use. There are several clinical adjuvants like emulsions (MF59 and Montanide) and Adjuvant Systems (AS03 and AS04), which have shown to induce potent antibody and T-cell response correlating to protection against various pathogens but have not been tested against leptospirosis (33–35). AS04 combines the TLR4 agonist monophosphoryl lipid A (MPLA), and aluminum salt is a new-generation adjuvant licensed for use in human vaccines (34). Human papilloma virus (HPV) vaccine and hepatitis B vaccine adjuvanted with AS04 induced superior antibody and T-cell response as compared to alum-adjuvanted vaccine (36). Emulsion-based adjuvants have been successfully used against various diseases and few adjuvants like MF59, ASO3, and Montanide adjuvants are approved for human use (35). Of these, Montanide ISA720VG is a new-generation water-in-oil emulsion adjuvant for human use. It has been used in human therapeutic vaccines in more than 200 clinical trials involving cancer, AIDS, malaria, or autoimmune diseases (33, 37).

In the present study, we formulated LAV in AS04 (LAV-AS04) and Montanide ISA720VG (LAV-M) and evaluated long-term immune response and generation of immunological memory compared with alum in the mouse model. Furthermore, we tested the protective efficacy of LAV-AS04 and LAV-M in the hamster model of leptospirosis.

## Results

### LAV-AS04 and LAV-M induced superior antibody and T-cell response compared to LAV-Alum

**Figure 1:**
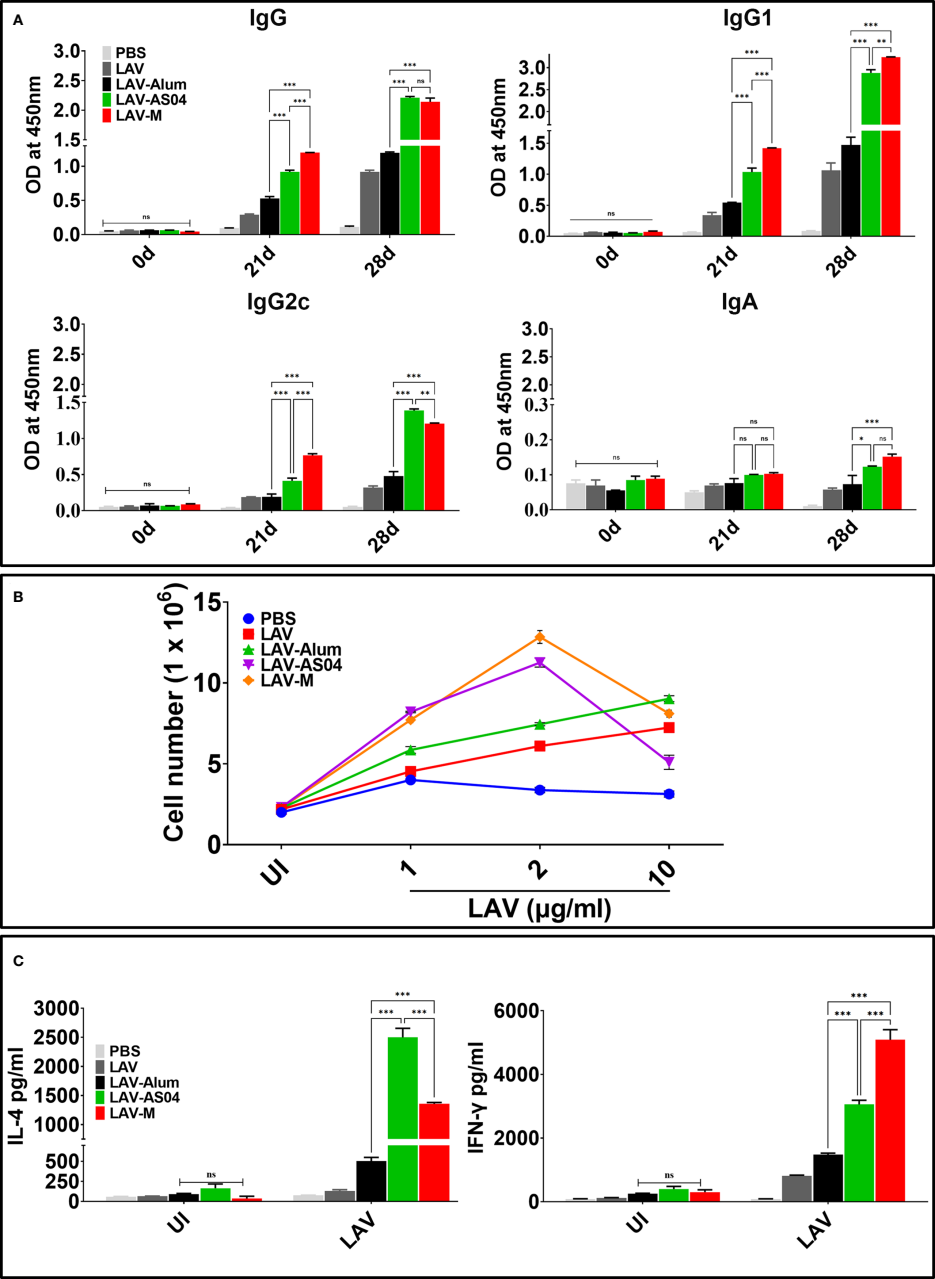
Humoral and cell-mediated responses mediated by LAV formulated in various adjuvants. Mice were immunized subcutaneously with PBS or LAV in alum or AS04 or Montanide on days 0 and 21 and then euthanized on day 28 to remove spleen to determine antigen-specific immune response. **(A)** Antibody response. LAV-specific antibody levels (IgG, IgG1, IgG2c, and IgA) at various time points (days 0, 21, and 28) analyzed by ELISA as detailed in *Materials and Methods*. **(B)** Lymphocyte proliferation. The splenocytes isolated from various immunized groups at day 28 were subjected to *in vitro* stimulation with recall antigen (LAV) and proliferation was assessed by counting the cells after 72 h. **(C)** Cytokine analysis. Splenocytes were stimulated with varying concentrations (1, 2, and 10 µg/ml) of LAV for 48–72 h and culture supernatant was analyzed for IL-4 and IFN-γ by using a sandwich ELISA kit following the manufacturer’s instructions. Data are representative of three different experiments. Significant differences were calculated using the one-way ANOVA (***, **, *, and ns indicate *p* < 0.001, *p* < 0.01, *p* < 0.05, and non-significant, respectively).

To test whether LAV formulated in AS04 or Montanide ISA720VG induced better humoral immune response than LAV-Alum, we analyzed the antibody levels in mice at 21 and 28 days post-immunization. Our results show that significantly higher levels of IgG was generated in animals immunized with LAV-AS04 and LAV-M than those immunized with LAV-Alum (**Figure 1A**). Both LAV-AS04 and LAV-M induced IgG1 but the level was significantly higher in the latter (**Figure 1A**). IgG2c was only detected in animals immunized with LAV-AS04 or LAV-M. Animals immunized with LAV-Alum or LAV without any adjuvant induced low levels of IgG and IgG1 and could not induce significant levels of IgG2c (**Figure 1A**). LAV formulated in any of the adjuvant failed to induce significant levels of IgA (**Figure 1A**). To analyze whether LAV formulated in AS04 or Montanide adjuvants can induce strong T-cell response, we stimulated splenocytes isolated from various groups with recall antigen LAV on day 28. Our result showed that splenocytes isolated from LAV-AS04 or LAV-M exhibited significantly higher (*p* < 0.05) levels of proliferation as compared to those from LAV-Alum (**Figure 1B**). Analysis of cytokines in culture supernatant showed that while alum induces Th2 response by inducing mainly IL-4 and low level of IFN-γ, AS04 and Montanide induced a mixed Th1/Th2 response with significantly higher levels of both IL-4 and IFN-γ (**Figure 1C**).

### LAV-AS04 and LAV-M induced generation of cytotoxic T cells

**Figure 2:**
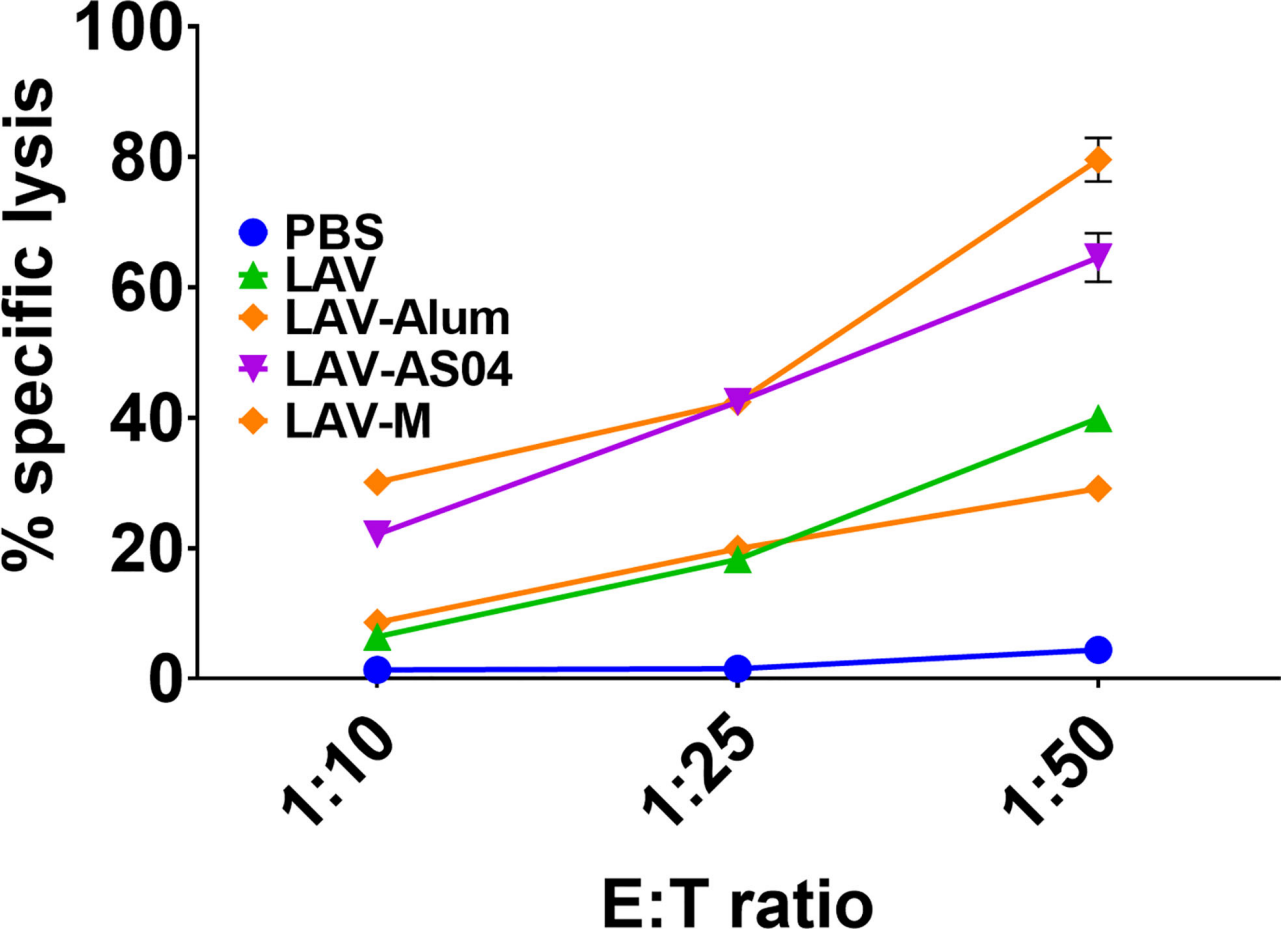
Cytotoxic T-cell response induced by various vaccines. Effector cells generated *in vitro* from splenocytes isolated from various groups were added to specific targets (DCs pulsed with LAV) in varying E:T ratios. Cytotoxicity was measured using a Cytotox kit (LDH method) following the manufacturer’s instructions. Data are representative of three different experiments.

To analyze whether LAV formulated in AS04 or Montanide adjuvants can induce production of cytotoxic T cells (CTLs), we isolated effector cells from splenocytes of animals immunized with LAV-Alum, LAV-AS04, or LAV-M and determined the lysis of target cells by using LDH assay. Our results show that a higher number of target cell (DCs stimulated with LAV antigen) lysis (70%–80%) was demonstrated by CTLs obtained from animals immunized with LAV-AS04 or LAV-M as compared to LAV-Alum or LAV (20%–30%) at the highest E:T ratio (**Figure 2**). Effectors obtained from both LAV-AS04 or LAV-M were not able to lyse non-specific targets (DCs stimulated with unrelated antigen OVA), further confirming the specificity of CTLs (data not shown). Thus, animals immunized with LAV formulated in AS04 or Montanide adjuvant were able to generate cytotoxic T cells.

### Strong adaptive response induced by LAV-AS04 and LAV-M correlated to the enhanced recruitment and activation of innate cells at draining lymph nodes

**Figure 3:**
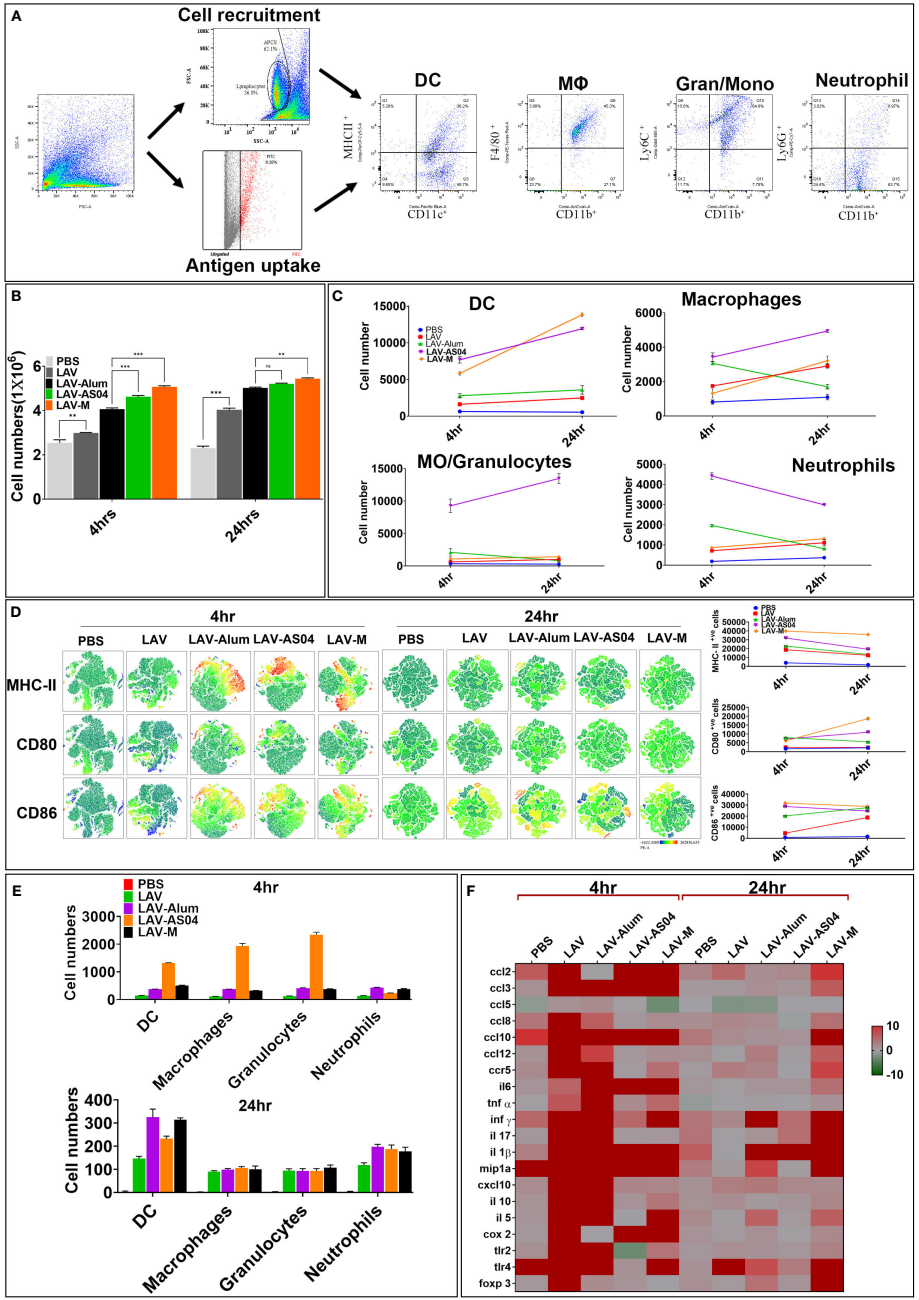
Analysis of innate immune cells at draining lymph nodes (DLNs) post injection of various vaccines. **(A)** Gating strategy. Schematic representation, gating strategy of different immune cells with specific markers recruited after the injection. **(B)** Analysis of total cells in DLNs. The total number of live cells in DLNs recovered at 4 and 24 h post-injection from individual mice were counted using the Trypan Blue dye exclusion method. **(C)** Analysis of type of cells recruited in DLNs. Cells recovered at 4 and 24 h post-injection were stained with fluorochrome-conjugated antibodies against specific markers and then analyzed by flow cytometry as described in *Materials and Methods*. **(D)** Analysis of activation status of cells recruited in DLNs. Cells recovered at 4 and 24 h post-injection were stained with fluorochrome- conjugated antibodies against specific markers (CD-80, CD-86, and MHC-II) and then analyzed by flow cytometry. The relative expression levels are shown for the indicated markers by tSNE analysis. **(E)** Analysis of antigen uptake by innate cells in DLNs. Uptake of Alexa Flour-488-labeled LAV by various innate cells at 4 and 24 h post-injection was analyzed by flow cytometry as described in *Materials and Methods*. **(F)** Inflammatory response at the site of injection. Injection site tissue were recovered 4 and 24 h post-injection, RNA was isolated and converted to cDNA, and gene expression was analyzed by RT-PCR as described in *Materials and Methods*. Data are representative of three different experiments. Significant differences were calculated using the one-way ANOVA (***, **, and ns indicates P < 0.001, < 0.01, and non-significant respectively).

Since adaptive immune response is driven by strong innate response, we analyzed adjuvant-induced recruitment of innate cells at draining lymph nodes (DLNs) at 4 h and 24 h post-injection. The cell numbers, type, and activation status were assessed by flow cytometry in pooled DLNs taken from individual mice (**Figure 3A**). Our results show that LAV-AS04 and LAV-M induced recruitment of a higher number of innate immune cells than LAV and LAV-Alum. LAV antigen without any adjuvant was also able to recruit a significantly higher number of cells as compared to the PBS group (**Figure 3B**). LAV-AS04 induced early recruitment (at 4 h) of a large number of DCs (CD11c+MCHII+), macrophages (CD11b+F4/80+), granulocytes/inflammatory monocytes (CD11b+Ly6C+), and neutrophils (CD11b+Ly6G+), which was significantly higher than LAV-Alum. While LAV-M was only able to induce early recruitment of DCs (CD11c+MCHII+), LAV-Alum recruited a large number of macrophages (CD11b+F4/80+) and significant levels of neutrophils (CD11b+Ly6G+), but failed to recruit significant levels of DCs or granulocytes/inflammatory monocytes (**Figure 3C**). We then tested the effect of these adjuvants in terms of their ability to cause activation and maturation of the recruited immune cells. Our result showed that LAV-AS04 and LAV-M caused significant activation and maturation as evident by enhanced expression of costimulatory molecules (CD80 and CD86) and maturation marker (MHC-II) at 4 h post-injection (**Figure 3D**). After establishing that LAV-AS04 and LAV-M generally induced a more powerful cell migration and activation compared to alum, the impact on antigen uptake was assessed. LAV labeled with Alexa Fluor™ 488 was used to enable tracking of antigen after delivery. LAV+ cells were rapidly detected in DLNs 4 h after injection in only LAV-AS04; however, these cells were detected at 24 h in all groups where LAV was predominantly taken up by DCs (**Figure 3E**). We then analyzed the ability of LAV-AS04 and LAV-M to induce expression of various cytokines, chemokines, their receptors, and adhesion molecules at the site of injection. Our gene expression analysis at 4 h and 24 h shows that LAV antigen without adjuvant modulated the expression of various cytokines/chemokines or their receptors, which peaked at 4 h, and then the level progressively declined at 24 h (**Figure 3F**). While AS04 modulated the expression at an early time point (4 h), LAV-M modulated expression at both 4 h and 24 h. PBS injection also induced basal level of expression of these cytokines/chemokines owing to injury due to needle at the site of injection.

### LAV-AS04 and LAV-M induced long-term immune response and generation of immunological memory

**Figure 4:**
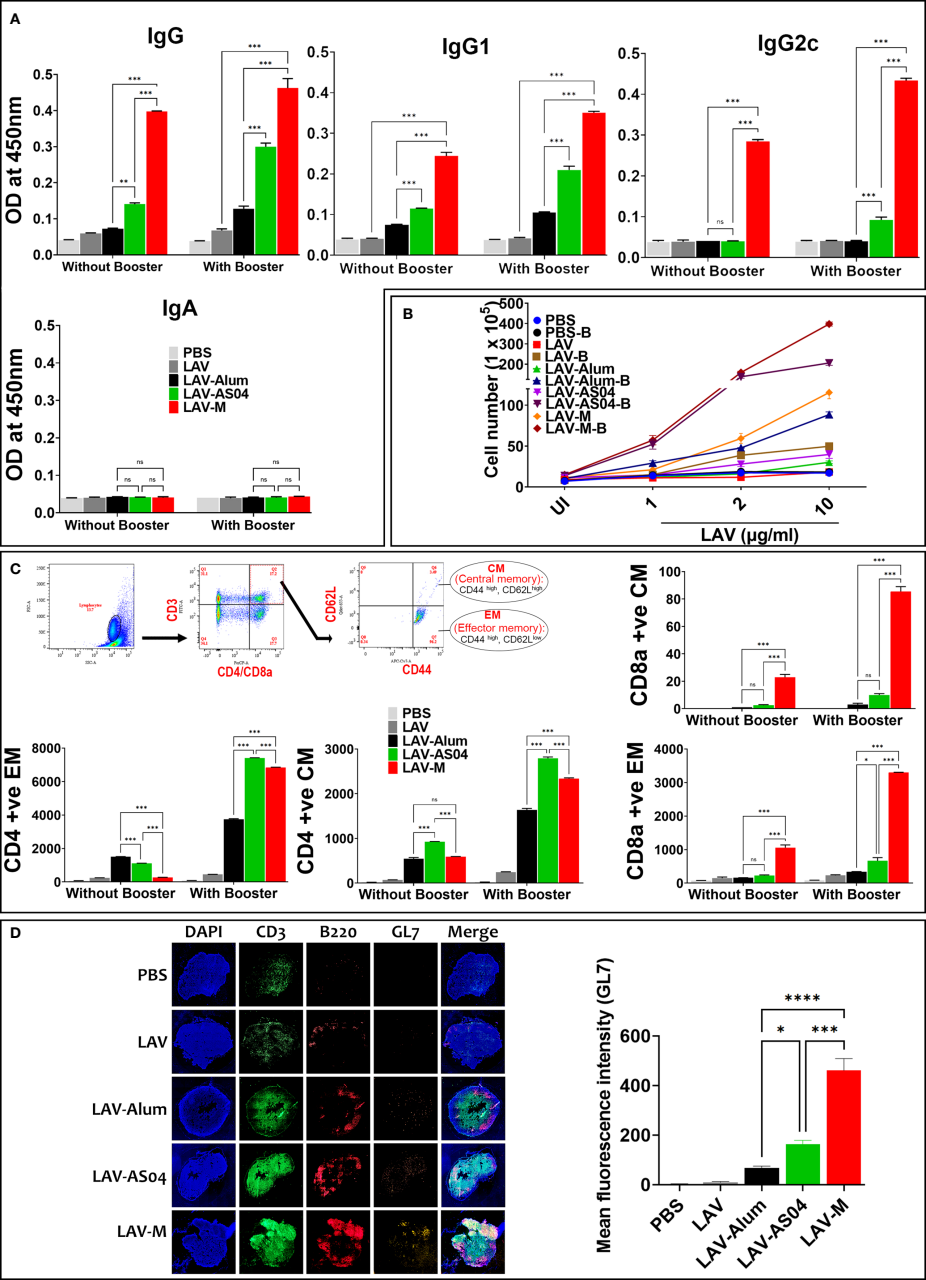
Evaluation of the long-term memory response induced by various vaccines. Mice were either bled and killed 6 months (day180) post-immunization or boosted and killed after 1 week (day 187) to analyze B-cell (antibodies) and T-cell memory response. **(A)** B-cell memory response. The antibodies including isotypes were analyzed in serum (obtained at day 180 and 187) by ELISA as described in *Materials and Methods*. **(B)** T-cell memory response. The splenocytes recovered from mice euthanized on days 180 and 187 were subjected to *in vitro* stimulation with recall antigen (LAV) and proliferation was assessed by counting the cells after 72 h. **(C)** Analysis of memory phenotype. Lymphocytes were stained with antibodies specific for CD3, CD4, CD8, CD62L, and CD44 and analyzed by flow cytometry. Representative flow cytometry plots show the gating strategy to analyze both central (CD44^high^ and CD62L^high^) and effector (CD44^high^, CD62L^low^) population of CD4 and CD8 T cells. The gated population obtained were plotted as histograms to show each phenotype. **(D)** Analysis of germinal centers. Draining lymph nodes (DLNs) taken from mice at 28 days post-immunization were processed as frozen tissue sections. Consecutive sections were stained with fluorochrome-conjugated anti-mouse CD3/CD45R/B220/GL-7 antibodies, mounted, and then examined under a fluorescent microscope using the tile scanning and stitched using the ZEN software as described in *Materials and Methods*. Data are representative of three different experiments. Significant differences were calculated using the one-way ANOVA (****,***, **, *, and ns indicates P < 0.0001, P < 0.001, <0.01, P < 0.05 and non-significant respectively).

The success of vaccines often depends on the induction of a strong and persistent memory response. To evaluate whether immunization with LAV-AS04 and LAV-M can induce long-term immune response with generation of immunological memory, we analyzed the antibody level in various groups 180 days post-immunization. Of various groups, only LAV-AS04 and LAV-M were able to induce long-term persistent response as significant levels of antibodies were detected in these groups even after 180 days post-immunization (**Figure 4A**). While LAV-AS04 was able to induce IgG and IgG1, LAV-M induced significantly higher levels of IgG and IgG1 with significant levels of IgG2c. To test the capacity of these adjuvants to elicit functional immune memory, we boosted the animals with a lower dose of non-adjuvanted recall antigen, and our results show enhanced level of antibodies in both AS04 and Montanide groups (**Figure 4A**). Animals immunized with LAV without adjuvant and LAV-Alum showed a subsequent drop in antibody titers and failed to evoke significant memory response following Ag boost (**Figure 4A**). To test the generation of memory T cells, we isolated the splenocytes and stimulated with recall antigen. Our results show a significantly higher level of lymphocyte proliferation in LAV-AS04 and LAV-M groups (**Figure 4B**). Moreover, lymphocytes isolated from animals 1 week after booster exhibited a significantly higher level of proliferation in both LAV-AS04 and LAV-M groups (**Figure 4B**). We then analyzed the memory phenotype of both CD4 and CD8 T cells characterized as CD44^high^ and CD62L^high^ (central memory, T_CM_) and CD44^high^ and CD62L^low^ (effector memory, T_EM_). Animals immunized with LAV-AS04 and LAV-M induced significantly higher levels of both central and effector memory CD4T cells as compared to alum (**Figure 4C**). However, only LAV-M was able to induce significant levels of both central and effector memory CD8T cells (**Figure 4C**). Animals immunized with PBS or LAV without adjuvant did not induce any significant level of memory T cells. Formation of germinal centers (GCs) is crucial for generation of long-lived plasma cells that secrete high-affinity antibodies and also for development of immunological memory. These GCs are formed in secondary lymphoid tissues like spleen and lymph nodes (specifically DLNs). To assess whether higher and long-term antibody response induced by LAV-AS04 and LAV-M correlates to their ability to modulate GCs, we isolated the inguinal lymph nodes (DLNs) from animals euthanized at 28 days post-immunization, and the presence of GCs was evaluated by identifying B220+GL-7+ cells by immunofluorescence. Our results show that LAV-AS04- and LAV-M-immunized mice developed more lymph node GCs than LAV-Alum as evident from the increased frequency of B220+GL7+ cells (**Figure 4D**).

### LAV-AS04 and LAV-M induced enhanced protective efficacy compared to LAV-Alum in the hamster model of leptospirosis

**Figure 5:**
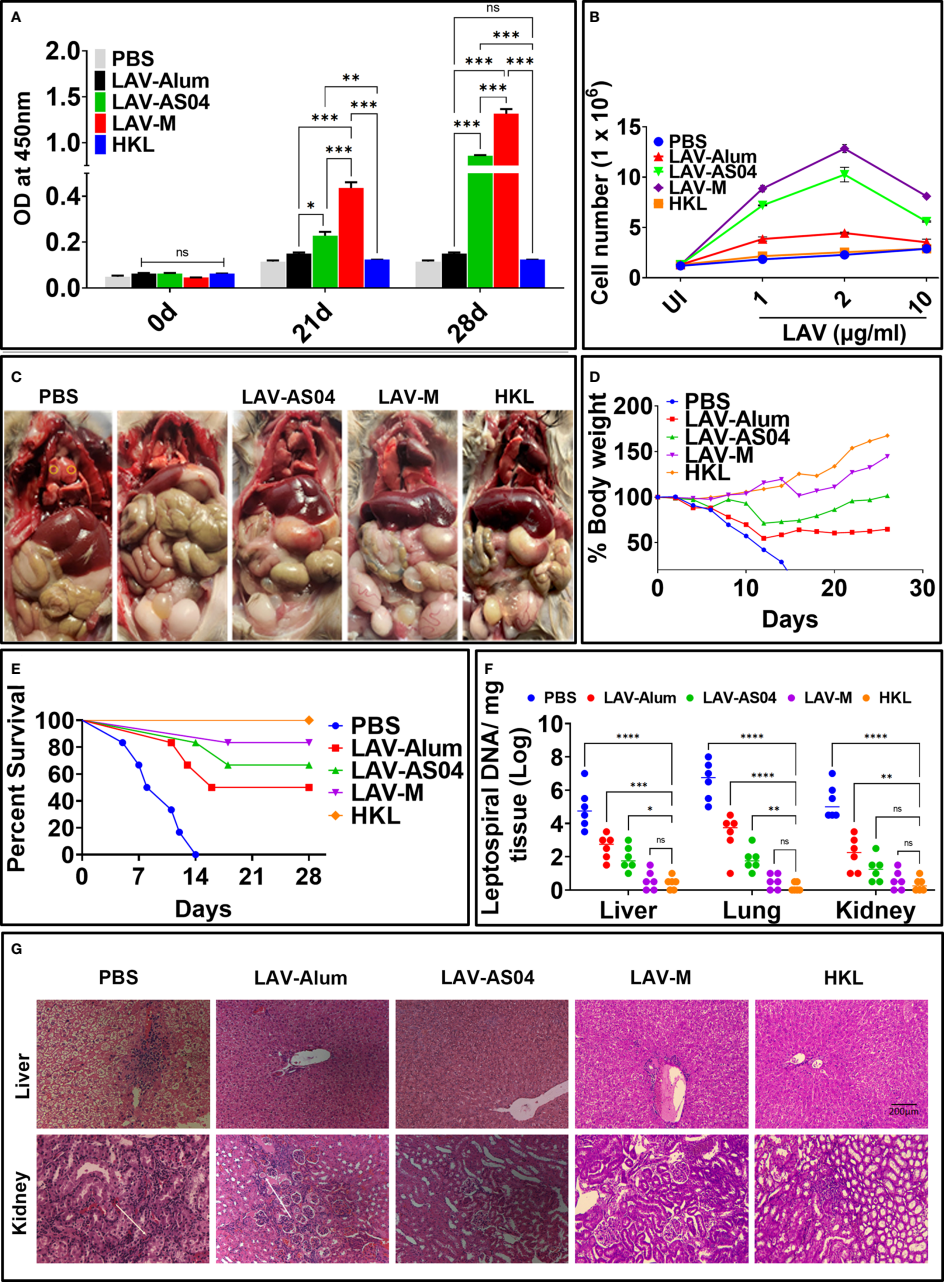
Evaluation of immune response and protective efficacy in hamster model of leptospirosis. Golden Syrian Hamsters (male, 4–5 weeks) were immunized subcutaneously with PBS or HKL or LAV in alum or AS04 or Montanide ISA720VG at day 0 and then boosted at day 21. **(A)** Antibody response. LAV-specific total IgG levels in serum (collected at 0, 21, and 28 days) analyzed by ELISA as described in *Materials and Methods*. **(B)** Cell proliferation. Splenocytes isolated from various immunized groups on day 28 were subjected to *in vitro* stimulation with recall antigen (LAV) and proliferation was assessed by counting the cells after 72 h. **(C)** Clinical manifestations in challenged animals. Gross appearance of the organs of the animals from various groups examined 28 days post-challenge with virulent *Leptospira*. **(D)** Bodyweight measurement. Challenged hamsters were weighed on a daily basis for 28 days and plotted as percent body weight as described in *Materials and Methods*. **(E)** Survival data. Kaplan–Meier’s plot showing percent survival of the immunized animals post challenge with virulent *Leptospira* and analyzed based on criteria described in *Materials and Methods*. **(F)** *Leptospira* burden. DNA was extracted from tissues (kidney, liver, and lungs) of the survived animals from various groups at 28 days post-challenge and subjected to RT-PCR to detect LipL32 and 16s using specific primers and then determine bacterial burden per milligram of tissue as described in *Materials and Methods*. Means are depicted as bold horizontal bars along with standard deviations. **(G)** Histopathology. Representative H&E staining of the liver and kidney sections was obtained 28 days after the challenge showing various pathological conditions. Data are representative of three different experiments. Significant differences were calculated using the one/two-way ANOVA (****, ***, **, *, and ns indicates P < 0.0001, P < 0.001, < 0.01, P < 0.05 and non-significant respectively).

Golden Syrian hamsters are the preferred and most widely used model for testing vaccines due to susceptibility to infection and reproducibility of results. Hence, we evaluated the immune response and protective efficacy of various vaccines in the hamster model of leptospirosis. The antibody response analyzed at days 21 and 28 (1 week after booster) shows significantly higher levels of IgG generated in animals immunized with LAV-AS04 and LAV-M (**Figure 5A**). Similarly, lymphocytes isolated from the LAV-AS04- or LAV-M-immunized group exhibited significantly higher (*p* < 0.05) levels of proliferation as compared to LAV-Alum (**Figure 5B**). Killed vaccine (HKL) was not able to generate LAV-specific antibodies and T cells (**Figures 5A, B**). To test if the immune response correlates to protection, we challenged animals at day 35 with virulent *Leptospira* and evaluated the protective efficacy in terms of progressive loss of body weight, survival (using endpoint criteria), histopathology, and bacterial load. The challenged animals in the non-vaccinated control group (PBS) showed typical signs of acute leptospirosis with necrosis and small foci of gross and microscopic pulmonary hemorrhage. While LAV-Alum and LAV-AS04 groups showed alleviated features, animals in LAV-M and HKL groups were close to normal (**Figure 5C**). As decline in body weight is considered as the earliest sign of progression of disease (leptospirosis), we included it as one of the clinical parameters and examined the body weight on a daily basis; ≥20% weight loss was considered as the endpoint criterion to prevent spontaneous death. Our results show that there was progressive weight loss in the control (PBS) group (**Figure 5D**). While LAV-Alum showed significant alleviation, the LAV-AS04-immunized group could significant delay this progression and then regained the weight 15 days post-challenge. In contrast, LAV-M-immunized animals showed a decline in weight 15 days post-infection for a very short window and quickly regained the weight whereas HKL showed no weight loss during the same period and increased their body weight until the end of the experiment (**Figure 5D**). The survival data on the 28th day post-infection showed 83% survival of the animals in LAV-M, 67% in LAV-AS04, and 50% in LAV-Alum, while none of the animals survived in the control PBS group (**Figure 5E**; **Supplementary Table 1**). Killed vaccine (HKL) provided 100% protection. Overall, there was a significant enhancement in the survival of animals vaccinated with LAV-M and LAV-AS04 as compared to the LAV-Alum group (**Figure 5E**). The leptospiral burden in the liver, lungs, and kidneys were measured by qPCR in terms of DNA copy number per milligram of tissue. While LAV-Alum-immunized animals showed a significant decrease in bacterial load, the burden was significantly higher than LAV-AS04 in all the organs (**Figure 5F**). The bacterial load in animals immunized with LAV-M was significantly lower than LAV-AS04 but similar to HKL. There was no significant difference in bacterial burden in all organs of animals vaccinated with LAV-M and HKL; however, HKL induced sterilizing immunity in more animals (**Figure 5F**). The histopathological analysis of organs (kidneys and liver) from various groups demonstrated varying degrees of lesions. PBS control animals showed severe kidney lesions characterized by marked chronic tubulointerstitial nephritis with severe atrophy, fibrosis, and infiltration of lymphocytes. Liver lesions in the PBS group were characterized by centrilobular necrosis with a lot of inflammatory foci (**Figure 5G**). Analysis of histopathological scores revealed that in the HKL group, 75% of animals were normal and 25% had mild lesions. Animals immunized with LAV-M (50% normal, 33% mild, and 17% moderate lesions) or LAV-AS04 (42% normal, 33% mild, and 25% moderate lesions) had significantly reduced lesions as compared to LAV-Alum. In the LAV-Alum group, only 25% of animals had mild lesions, 50% of animals had moderate lesions, and 25% of animals were severely affected (**Figure 5G**; **Supplementary Table 2**).

## Discussion

The currently available killed vaccine for leptospirosis is used mainly in animals and suffers from several limitations like toxicity, short-term and serovar-specific immunity, and inability to provide sterilizing immunity (8). The immune response generated against natural infection or killed vaccine induces protection, which is primarily mediated by antibodies against LPS. Since LPS is a T-independent antigen, there is a low, serovar-specific, and short-term response, which lacks immunological memory. Thus, the vaccine based on T-dependent protein antigens that are conserved among different serovars and can induce long-term humoral and T-cell response is urgently needed (38). In recent years, major focus has been on the development of subunit vaccines based on protein antigens that are conserved among different pathogenic serovars (39). Several surface proteins have been identified, which, when used in recombinant form with adjuvant, have shown to induce significant levels of protection against challenge in the hamster model of leptospirosis (9–18). Of these proteins, *Leptospira* immunoglobulin-like proteins (LigA and LigB) are the most promising subunit vaccine candidate (19, 20, 22, 25, 26, 28). LigA is not present in all pathogenic species of *Leptospira*, but has been found in only *L. interrogans* (serovar Lai is an exception) and *L. kirschneri* (40). LigA, specifically its variable C-terminal region (LAV), has been demonstrated to be a very good vaccine candidate in different platforms like subunit protein, DNA, encapsulated, lipidated, and carbon nanotubes conferring high level of protection (efficacy varying from 60% to 100%) against challenge in animal model as tested by several groups (19–26, 41–44). Although the protective role of LigA is well established, recent studies showed that mutants of LigA and LigB (LigAB) are attenuated in virulence and Lig proteins are involved in serum resistance of pathogenic *Leptospira*, further indicating that these proteins are an important target and anti-LigA immune response can be protective (45, 46). Adjuvants have been key to the success of these subunit vaccines, and several adjuvants have been evaluated to enhance the immune response and efficacy of these protective antigens including LigA (8, 17, 19, 21, 22, 28, 31, 32). While alum was most widely used, it provided partial protection, and Freund’s adjuvant being very potent has been associated with reactogenicity, pain, and distress to animals (10, 20, 24, 28–30, 47). Moreover, none of the vaccine formulations including these adjuvants provided sterilizing immunity or prevented renal colonization of *Leptospira* (9–18). Additionally, these adjuvants are non-clinical, which is a major hurdle in further refining them to be used in human or animal vaccination. Recently, several clinical adjuvants have become available but have not yet been evaluated along with subunit antigens of *Leptospira* (48). The choice of adjuvant included in vaccine depends on the type of protective immune response desired against a particular infection. Although correlates of protective immunity against *Leptospira* is not very well understood, it is believed that antibodies play a major role in protection although they themselves are not sufficient to eliminate the bacteria (49, 50). Furthermore, inferences from several studies in small and large animals like cattle conclude that apart from antibodies, CD4 or γδ T cells producing IFN-γ play a major role in inducing protective immune response (51–53). Moreover, the presence of *Leptospira* in monocytes/macrophages and/or Vero cells indicate that it could have a short intracellular phase that might help in immune evasion (54–56). Thus, an ideal vaccine should include adjuvant that is capable of inducing a strong antibody response (Th2 response) as well as a significant level of IFN-γ (Th1 response) for killing leptospires residing in immune cells. Since innate response activated specifically through TLR2 and TLR4 plays a major role in protection against *Leptospira*, we speculated that a vaccine or adjuvant formulation having TLR4 agonist like Monosphosphoryl lipid A (MPLA) might induce strong innate and subsequent adaptive response (49, 57). MPLA from *Bordetella pertussis* in combination with chimeric protein rChi, which includes LAV in its sequence, has shown to induce protection against challenge with virulent *Leptospira* in an animal model (58). AS04 (alum + MPLA) is a clinical adjuvant that has shown strong immune response correlating to better efficacy than alum against various infections in animal models and humans (33–36). Emulsion-based adjuvants have been successfully used against various diseases and few adjuvants like MF59, Montanide adjuvants, and ASO3 are approved for human use. Freund’s adjuvant, which is a water-in-oil adjuvant capable of generating a balanced immune response, is good for initial screening; however, severe toxicity has precluded its use in clinical studies. To overcome this toxicity issue, non-metabolizable mineral oil (paraffin oil) was replaced with metabolizable oil squalene and modified water-in-oil Montanide adjuvants (ISA51, ISA720VG, etc.) were developed by Seppic, France. Montanide adjuvants have shown to generate enhanced humoral and cellular immune response to malaria vaccine compared to alum and also did not show any serious side effects (33, 37, 59).

Keeping these reports in view in the present study, we evaluated the immune response and protective efficacy of LigA (LAV) formulated in clinical adjuvants AS04 and Montanide ISA720VG and compared with the widely used alum. Due to the unavailability of hamster-specific reagents, we used mice to evaluate immune response and decipher the mechanism of action of these adjuvants. Our results show that LAV formulated in AS04 or Montanide adjuvant induced significantly higher levels of antibodies than LAV-Alum. Both LAV-AS04 and LAV-M induced significantly higher levels of T-cell proliferation and induction of both IL-4 and IFN-γ. While LAV-Alum induced a Th2 response, LAV-AS04 and LAV-M induced a balanced or mixed Th1/Th2 response (**Figure 1**). Thus, low to moderate levels of protection induced by various subunit vaccines against Leptospira formulated with alum and other adjuvants may be attributed to their inability to induce a balanced or mixed Th1/Th2 response. These results also correlate to the previous observation of association of these responses with enhanced protective efficacy in a hamster model (10, 28–30). Although generation of CD8T cells, specifically cytotoxic T lymphocytes (CTLs), is important against intracellular pathogens, the existence of cytotoxic CD8T cells in leptospirosis patients has been previously reported (60). Furthermore, these CD8T cells were able to elicit specific CTL responses when stimulated with the LigA peptide. Our results show that immunization with LAV-AS04 and LAV-M induced the generation of CTLs capable of killing 70%–80% target cells, highlighting the importance of these adjuvants and their ability to generate CD8T cells, specifically CTLs (**Figure 2**).

Vaccine antigen is first recognized by local and infiltrating innate immune cells at the injection site and DLNs; hence, the unique repertoire of these cells may greatly influence the adaptive immune responses (61). Several adjuvants have shown to modulate the expression of chemokines, cytokines, or their receptors at the site of injection (62). Our results show that both LAV-AS04 and LAV-M were able to recruit more innate immune cells and cause a significant level of activation and also modulate the expression of several cytokines, chemokines, and receptor genes (**Figure 3**). AS04 is known to induce local inflammatory response leading to recruitment of DCs and monocytes (63). Several emulsion adjuvants have shown to be capable of recruiting innate cells, which is greatly influenced by their droplet size (64). Thus, our results are in agreement with previous studies demonstrating the ability of adjuvants to recruit innate cells and traffic the antigen to the DLNs to induce antigen-specific adaptive immune responses (65, 66).

Generation of immunological memory against *Leptospira* is very important as natural infection or vaccination does not seem to generate memory T cells that can be activated by *in vitro* stimulation (67). Although several subunit vaccines (containing different adjuvants) have been tested for immune response and protective efficacy, no attempt has been made to evaluate the generation of long-term response or check immunological memory generated by these adjuvanted vaccines. Our results show that both LAV formulated in AS04 or Montanide generated strong B- and T-cell memory (**Figure 4**). Although AS04 induced strong CD4 T-cell memory, Montanide was able to induce memory CD8 T cells as well. Moreover, no skewing of the Central vs. Effector memory populations in either CD4 or CD8 T cells was observed regardless of the adjuvant being used. The formation of GCs is a critical element for the production of high-affinity antibodies and long-lived plasma cells, and the generation of immunological memory (68). It has been demonstrated that high antibody titers without GC reaction is not protective, suggesting that GCs are critical for long-term vaccine efficacy (69). Our results clearly show that both AS04 and Montanide were able to form GCs as indicated by the higher number of GL7+ B cells; however, the GC reaction was significantly higher in LAV-M (**Figure 4**). The ability of emulsion adjuvants like Montanide to enhance GC reaction is multifactorial and may be attributed to the increased availability of antigen, the selective polarization of naïve T cells to T_FH_ cells, and the modulation of memory B-cell formation. Additionally, the slow degradation of oil coupled with the ability to retain antigen by emulsion adjuvants is also critical for GC reaction and maintenance of long-term memory (70).

To check if the immune response in mice correlates to protective efficacy, we used the hamster model of leptospirosis. Our results show that hamsters immunized with LAV-AS04 or LAV-M induced significantly higher levels of antigen-specific antibodies and enhanced levels of T-cell proliferation than those immunized with LAV-Alum (**Figure 5**). The immune response correlated to a higher level of protection as evident from enhanced survival, and reduced bacterial load and lesions in vital organs (**Figure 5**; **Supplementary Tables 1**, **2**). To be more consistent with survival data, we used endpoint criteria to assess the survival of the challenged animals due to inconsistency in getting 100% lethality and also unexpected recovery of the control group. Interestingly, LAV formulated in AS04 adjuvant induced a much higher level of protection (67%) with sterilizing immunity in some animals as compared to only 60% in previously tested MPLA containing the LMQ adjuvant (71). Furthermore, this efficacy was achieved with only two doses of LAV-AS04 as compared to three doses of LAV-LMQ. LAV-M imparted protective efficacy similar to HKL with sterilizing immunity in some of the animals (**Figure 5**). Although HKL induced the best protective efficacy, it was not contributed by LAV-specific antibodies and T cells correlating to previous reports of lack of expression of LigA in bacteria cultured *in vitro* (72, 73).

**Figure 6:**
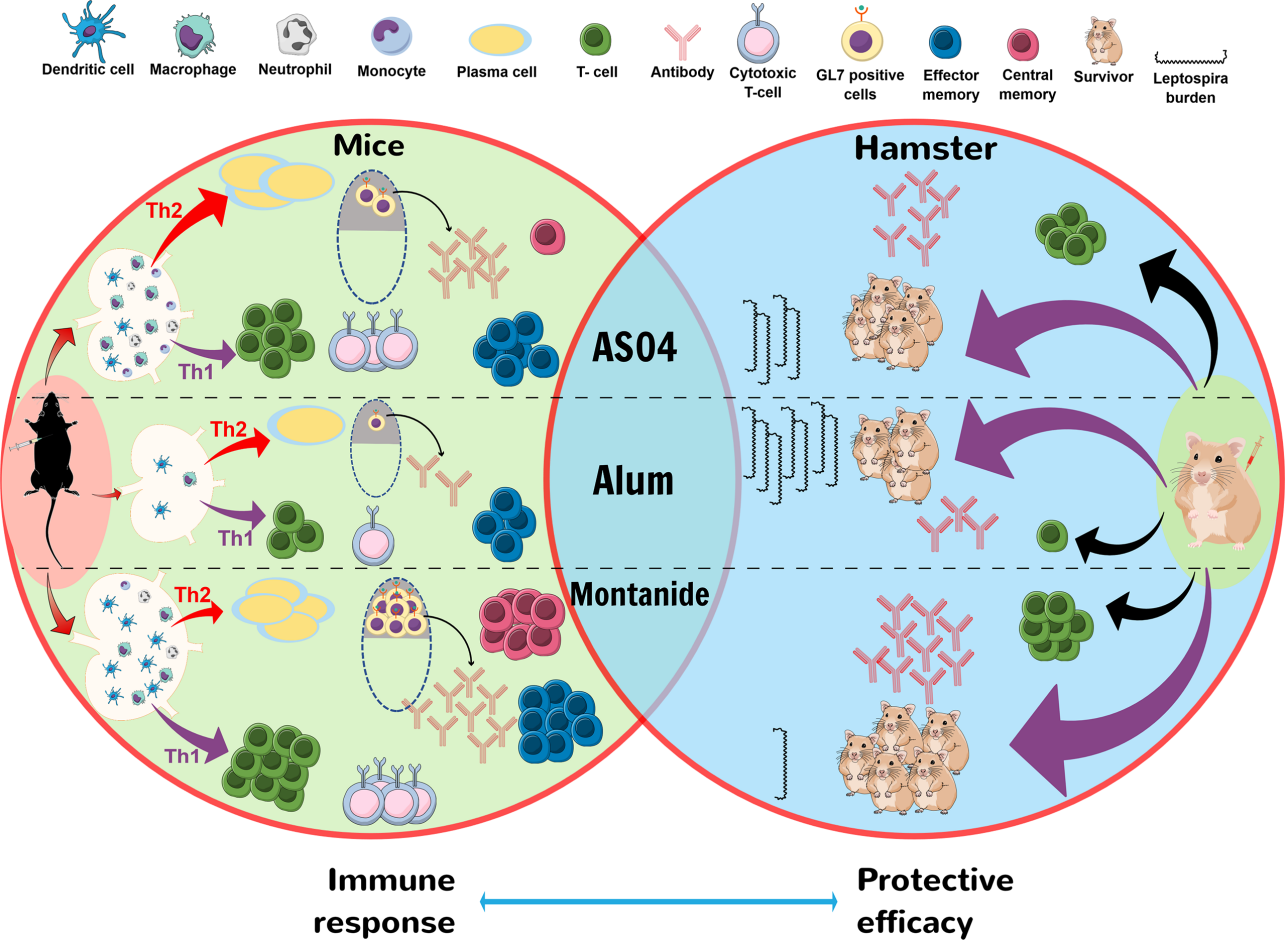
Schematic presentation of mechanism by which LAV-AS04 or LAV-M induces superior immune response and protective efficacy than LAV-Alum in mice and hamster model. Mice immunized with LAV-Alum were able to recruit few innate immune cells at DLNs leading to induction of low level of B cells (antibodies), T cells (mainly Th2 cells), low GC reaction, and limited immunological memory (low numbers of effector memory cells). In contrast, LAV-AS04 recruited a significantly higher number of innate immune cells leading to induction of enhanced level of antibodies and T cells (both Th1 and Th2 cells) and also generation of both effector and central memory T cells. LAV-M recruited a much higher number of innate immune cells correlating to higher level of antibodies and T cells than LAV-AS04, along with enhanced GC reaction and generation strong memory response. Hamsters immunized with LAV-M and LAV-AS04 generated significantly higher levels of antibodies and T cells than LAV-Alum. These antibodies and T cells were protective and were able to kill the *Leptospira* leading to reduced bacterial load in organs.

In conclusion, our study demonstrated that LAV formulated in clinical adjuvants AS04 and Montanide ISA720VG induced superior immune response compared to alum, which may be attributed to their ability to enhance recruitment and activation of immune cells leading to induction of strong innate and subsequent adaptive immune response. Furthermore, the strong immune response correlated to the enhanced protective efficacy in the hamster model of leptospirosis (**Figure 6**). Thus, both AS04 and Montanide ISA720VG are promising adjuvants and can be further tested against leptospirosis in clinical trials.

## Materials and methods

### Animals

Male C57BL/6 mice (5–6 weeks) were obtained from the Animal Resource and Experimental Facility of the National Institute of Animal Biotechnology (NIAB), Hyderabad. Mice were kept under standard specific pathogen-free conditions and received water and food *ad libitum* at the facility. Male Golden Syrian hamsters (4–5 weeks old), originally procured from the Jackson Laboratory, USA, was maintained at Jeeva Life Sciences Pvt. Ltd., Hyderabad, and experiments were performed in their facility. All the animal procedures were performed according to the norms of the Institutional Animal Ethics Committee (IAEC) regulated by the Committee for the Purpose of Control and Supervision of Experiments on Animals (CPCSEA) of India.

### Chemicals and reagents

Cell culture reagents were procured from Sigma-Aldrich (USA) unless otherwise mentioned; ELISA kits were procured from R&D Biosystems. Flow cytometry reagents and antibodies were from BD Biosciences and Santa Cruz, respectively, unless specified. Monophosphoryl Lipid-A and Alhydrogel<sp>® adjuvant 2% (alum) were purchased from *In vivo*gen. Montanide ISA720VG, originally procured from SEPPIC, France, was a kind gift from Dr. S. N Singh, Biovet Pvt. Ltd.

### Preparation of antigen and vaccine formulation

The LAV was purified as described previously (74). Briefly, BL21(DE3) carrying the expression plasmid (pET28a-LAV) were grown at 37°C overnight on LB broth containing 50 μg/ml kanamycin, and the expression of the protein was induced with 1 mM isopropyl β-D-1-thiogalactoside (IPTG). The cells were harvested by centrifugation at 10,000 rpm, and the cell pellet was resuspended in 100 mM Tris-HCl and 150 mM NaCl, pH 8.0, followed by sonication at constant pulses. The lysate was centrifuged to remove cell debris, and the supernatant was subjected to affinity chromatography using Ni-NTA beads column (Takara). Eluted protein was dialyzed against 1×PBS with four changes for 2 days at 4°C. The protein was then passed through Detox-Gel (Pierce, USA) to remove any contaminating LPS from *E. coli*, and a residual trace amount of LPS was monitored by Limulus amoebocyte lysate (LAL, Endotoxin Detection Kit, Pierce, Thermo, USA) assay following the manufacturer’s instructions. The endotoxin level as determined by LAL assay was found to be <0.012 EU/ml. The purified protein was checked for size and purity by SDS-PAGE, and concentration was estimated using the Bradford reagent (Sigma, USA).

### Immunization

Mice (10 animals/group) were immunized subcutaneously with various vaccine formulations as detailed in **Table 1**.

**Table 1:** Vaccine formulation and immunization schedule.

Group	Antigen (µg/50 µl)		Adjuvant (µl)	Volume (µl)
	Immunization	Booster		
PBS	0	0	–	100 µl
LAV	10	5	–	100 µl
LAV-Alum	10	5	Alum 50 µl	100 µl
LAV-AS04	10	5	Alum 50 µl + MPLA 5µg	100 µl
LAV-M	10	5	Montanide ISA 720 50 µl	100 µl

### ELISA

Serum samples from individual mice were collected at various time points and total antibody or isotypes (IgG, IgG1, IgG2c, and IgA) were evaluated by ELISA using standard protocol. Briefly, 96-well microtiter plates (Nunc, Denmark) were coated with LAV (100 ng/well) in 0.1 M bicarbonate buffer and incubated overnight at 4°C. The plates were washed three times with 1 × PBS containing 0.05% Tween 20 (PBST). The plates were then blocked with 1% BSA for 2 h at room temperature. After the usual washing steps, 100 µl of serum (control and vaccinated) diluted 1:100 to 1:100,000 in PBST was added to each well and incubated for 1 h at 37°C in a humid chamber. The plates were washed three times with PBST and incubated with 100 µl of a 1:6,000 (Southern Biotech) dilution of HRP-conjugated goat anti-mouse IgG or IgA for 30 min at room temperature. For isotype determination, both HRP-conjugated anti-mouse IgG1 and IgG2c were added in separate wells with specific dilutions (1:6,000, Southern Biotech) and incubated for 30 min. After washing the plates five times with PBST, 100 µl of TMB substrate was added to each well. The plates were incubated in the dark at room temperature for 20 min. The enzymatic reaction was stopped by the addition of 2N H_2_SO_4_, and the optical density was read at 450 nm by using an ELISA reader.

### Cell proliferation and cytokine estimation

Animals were euthanized on the 28th day post-immunization, and splenocytes were prepared from each group using standard procedure. Spleens pooled from three to four mice were homogenized and cells were pelleted down by centrifugation at 1,500 rpm for 5 min. RBCs were lysed using ACK lysis buffer (Invitrogen, USA), and the cells were resuspended in wash buffer (PBS with 2% FCS). Cells were centrifuged at 1,500 rpm for 5 min, resuspended in RPMI supplemented with 10% fetal bovine serum (Invitrogen), 100 U/ml penicillin, and 100 mg/ml streptomycin, and counted using the trypan blue exclusion method. Splenocytes (1×10^5^cells/well) were seeded in a 24-well plate and induced with varying concentrations of LAV (1, 2, and 10 µg/ml) for 48 to 72 h. Cells were recovered by gentle pipetting and counted. In another set of experiment, culture supernatant was collected and cytokine levels of IL-4 and IFN-γ were estimated using sandwich ELISA kits (R&D Systems) as per the manufacturer’s instructions.

### Generation of bone marrow-derived DCs

Bone marrow-derived dendritic cells were prepared as described previously (32). Briefly, bone marrow recovered from femur and tibia of mice was passed through a 70-µm cell strainer in sterile culture dish containing complete DMEM medium. The cell suspension was centrifuged at 1,000 rpm for 5 min, and the supernatant was discarded. The RBCs were lysed by ACK lysis buffer and washed three times and finally suspended in complete medium. 10^7^ bone marrow cells per well were cultured in six-well plates in 4 ml of complete DMEM medium supplemented with GM-CSF (20 ng/ml) and IL-4 (5 ng/ml) (Peprotech). On days 2 and 7, half of the medium was replaced with new medium supplemented with GM-CSF (40 ng/ml) and IL-4 (10 ng/ml). On day 9, non-adherent and loosely adherent cells were removed. The adherent cells were then harvested by gentle washing with PBS, pooled, and used for specific assays.

### CTL assay

T-enriched cells were prepared from spleens of various immunized groups, and cytotoxicity assay was performed using the LDH method as described previously (32, 75). Briefly, 5×10^5^ DCs (prepared as described above) were pulsed with LAV. For the last hour of culture, mytomycin C (50 μg/ml for 45 min) was added. A total of 3×10^7^ spleen cells were added in 10 ml of RPMI plus 10% FBS containing 0.2 ng/ml IL-2, in 25-cm^2^ tissue culture flasks, kept upright. After 5 days at 37°C in a humidified atmosphere supplemented with 5% CO_2_, the non-adherent cells were recovered from the flask and used as effector cells (E). The cells were counted and incubated with DCs pulsed with LAV (specific target) or DCs pulsed with OVA (non-specific target). The reaction mixture was set up with varying E:T ratios (10:1, 25:1, and 50:1) for 5 h at 37°C in a humidified atmosphere supplemented with 5% CO_2_, and lysis of target cells was determined using a non-radioactive cytotoxicity assay kit (Cytotox 96, Promega) following the manufacturer’s instructions. Specific target cell lysis by CTLs was calculated as the percentage of total LDH activity of target cells as follows: % specific lysis = (experimental release − spontaneous release)/(maximum release – spontaneous release).

### Flow cytometry

The antigen (LAV) was labeled with the Alexa Fluor™ 488 (Invitrogen-A10235) as per the manufacturer’s instructions to assess the antigen uptake in the cell recruitment studies. Cells from DLNs isolated at various time points (4 h and 24 h) were washed and Fc receptors were blocked with anti-mouse CD16/CD32 antibody in FACS buffer at 4°C. The cells were stained with anti-mouse CD11c and MHC-II (DCs), CD11b/F4/80 (Monocytes/Macrophages), CD11b/Ly6C (Monocytes), CD11b/Ly6G (Neutrophils), CD80, CD86, and MHC-II for analysis of specific cell type and their activation status. Splenocytes isolated from immunized mice of various groups were stained with anti-mouse CD3, CD4, CD8, CD44, and CD62L. The cells were washed and fixed with 1% paraformaldehyde. A total of 50,000 or 100,000 events were acquired depending on type of experimental sample using a BD FACS Fortessa. All the data were analyzed by using FlowJo software (Tree Star Inc.).

### Lymph node sectioning and immunofluorescent staining

Lymph nodes were obtained from various groups, sectioned, and stained as described previously (76, 77). Briefly, lymph nodes were placed in 10 ml of 4% paraformaldehyde (PFA) for a 1h duration and then transferred into 30%, 20%, and 10% sucrose solutions gradually for dehydration at 4°C. Lymph nodes were frozen in OTC in cryomold, by Tissue-Tek using liquid nitrogen. Frozen lymph node samples were stored in a −80°C fridge. Sections were obtained at a thickness of 12 μm using a Leica Cryostat (Leica Geosystems, Heerbrugg, Switzerland). After rehydration in Tris-buffered saline and blocking in Tris-buffered saline with 5% BSA and 0.05% Tween 20, the sections were stained with FITC-conjugated rat anti-mouse CD3 (555274, BD Biosciences, 1:300), Per-CP-conjugated CD45R (B220), Anti-mouse (130-102-815, Miltenyi Biotec, 1:100), and PE-conjugated T- and B-Cell Activation Antigen (GL-7) Rat Anti-Mouse (561530, BD Biosciences, 1:200). Slides were incubated overnight at 4°C in a moist chamber. Stained slides were mounted with VECTASHIELD<sp>® Antifade Mounting Medium with DAPI (Vector laboratories) to stain the nucleus. Immunofluorescence-stained lymph node sections were imaged using a CarlZeiss Axio scope VII microscope with 20× Plan Apochromat 0.45 NA objective and EXFO X-Cite metal halide light source. Images were captured using Zen software by tile scanning mode with a Hamamatsu ORCA-ER CCD camera and processed (stitched).

### RT-PCR

Injection site tissues were taken out after various time points and stored in 500 µl of TRIzol (Invitrogen, Carlsbad, CA) and equal volumes of chloroform were added; samples were centrifuged at 12,000 rpm for 5 min at 4°C. The aqueous phase was then passed through RNA easy mini columns (MN) and RNA was purified following the manufacturer’s protocol. RNA quality was checked by running on a formaldehyde gel for 16s and 18s RNA bands and also on Bioanalyser. The RNA quantity was assessed by UV spectroscopy and purity by 260/280 ratio. First-strand cDNA was synthesized using PrimeScript 1st strand cDNA Synthesis Kit (Takara) following the manufacturer’s instructions. RT-PCR was performed in 96-well microtiter plates on a Bio-Rad sequence detection system. The two-step amplification was performed in a 10-µl reaction volume containing 50 ng of cDNA, 10 µM each primer (**Supplementary Table 3**) and SYBR green (Bio-Rad). Samples were run in triplicate and data were analyzed with the Sequence Detection System (Bio-Rad). The experimental data were presented as fold changes of gene expression of stimulated cells at various time points relative to control. mRNA levels of the analyzed genes were normalized to the amount of β-actin or GAPDH present in each sample. All primers were synthesized by IDT and their sequences are given in (**Supplementary Table 3**).

### Infection experiments

*Leptospira culture*—The virulent *L. interrogans* serovar Pomona was cultured at 28°C under aerobic conditions in liquid Ellinghausen-McCullough-Johnson-Harris (EMJH) medium (Difco, BD, USA) supplemented with 10% (vol/vol) EMJH enrichment medium (BD, USA). Virulent Leptospira were routinely maintained by iterative passage in golden Syrian hamsters and subsequent isolation from kidneys.

*Immunization and challenge—*Male hamsters (4–5 weeks old) were immunized subcutaneously with PBS, Heat killed bacterin (10^9^), or LAV (50 μg/animal) in alum or AS04 or Montanide in a 200-μl volume. Animals were boosted with 25 µg of antigen/animal on day 21. Prior to immunization, animals were anesthetized by intraperitoneal injection of 100 µl of ketamine (10 mg/ml)/xylazine (1 mg/ml) per 130 g of body weight. Two weeks after booster (day 35), animals were challenged intraperitoneally with 100 × ED_50_ of virulent *L. interrogans* serovar Pomona. ED_50_ was determined as described previously (78). Endpoint criteria included hematuria, loss of appetite, gait or breathing difficulty, ruffled fur, hunched posture, prostration, or weight loss of >20%. The clinical signs were observed thrice a day for 4 weeks and the animals showing serious clinical signs (moribund) were euthanized after blood collection and counted as dead. The hamsters that survived the challenge were bled and sacrificed at the end of observation. The kidneys, liver, and lungs were collected aseptically for determining bacterial load and histopathology.

*Immune response*—Antibody (IgG) level in sera on days 0, 21, and 28 were determined by ELISA as described elsewhere (22, 28). Briefly, LAV (200 ng/well) in 0.1 M bicarbonate buffer was coated on polystyrene plates at 4°C overnight. After the usual steps of blocking and washing, hamster sera (ranging from 1:100 to 1:100,000) were added and further incubated for 1 h at RT. After the usual steps of washing, anti-hamster total IgG (1:6,000, Southern Biotech) was added and further incubated for 30 min at RT. The wells were washed, followed by the addition of 100 µl/well of TMB, and then after 20 min, 50 μl of 2N H_2_SO_4_ was added, and plates were read at 450 nm. Lymphocyte proliferation was determined by stimulating splenocytes from various groups with LAV (1, 2, and 10 µg/ml) and counting cells after 48–72 h.

*Determination of bacterial load—*Bacterial load was determined in organs of all the infected animals whenever they reached the endpoint criteria and also for those that did not reach the endpoint criteria and survived till the end of experiment. Bacterial load was determined using quantitative RT-PCR method as described previously (79). Briefly, tissues (kidney, liver, and lung) were sliced into small pieces and total DNA was extracted using the standard protocol. The PCR was performed using 2× SYBR Green PCR Master Mix (Bio-Rad) with specific primers for leptospiral 16s rRNA and LipL32 on a Bio-Rad Real-Time PCR System according to the manufacturer’s instructions. Leptospiral DNA standard curve was constructed from 10-fold serially diluted DNA of *L. interrogans* serovar Pomona equivalent to 2 × 10^1^ to 2 × 10^9^ cells/ml.

*Histopathology—*Hamster tissues were collected and fixed by immersion in 10% neutral buffered formalin. The fixed tissues were sectioned at 5 μm, stained with hematoxylin and eosin, and examined by light microscopy. The severity of *Leptospira*-induced lesions was graded by a board-certified veterinary pathologist who was blinded to the treatment group. Tubulointerstitial nephritis was assessed as 0 = normal, 1 = mild, 2 = moderate, and 3 = severe, using criteria as previously described (28). Liver pathology was graded based on the average number of inflammatory foci in 10× fields: 0 = normal, 1 = 1–3, 2 = 4–7, and 3 = >7.

### Statistical analysis

For most of the experiments, one-way analysis of variance (ANOVA) using Dunnett hypothesis test was executed to analyze the results, unless otherwise mentioned. The data were represented as the mean of triplicates ± SEM. *p* < 0.05 was considered as significant.

## Data availability statement

The data that support the findings of this study are available from the corresponding author upon reasonable request.

## Ethics statement

The animal study was reviewed and approved by Institutional Animal Ethics Committee (IAEC) of NIAB regulated by the Committee for the Purpose of Control and Supervision of Experiments on Animals (CPCSEA) of India.

## Author contributions

SMF conceived the idea and designed the experiments. VPV, MK, AK, and SK performed the experiments. VPV, MK, AK, SK, and SMF analyzed the data. VPV and SMF wrote the initial draft and SMF edited the manuscript. All authors approved the final version of the manuscript.

## Acknowledgments

This work was supported partly by the DST-SERB-funded project EMR/2017/002468 (SP046) on developing *Leptospira* vaccines and partly by the DBT-NIAB flagship project No. BT/AAQ/01/NIAB-Flagship/2019 (SP051) on host–pathogen interaction (for SMF), which are funded by the Department of Biotechnology, Ministry of Science and Technology, Government of India. Financial support from the NIAB core fund is duly acknowledged. The authors would like to thank the Director, NIAB for providing the necessary infrastructural facility and support for the execution of the above study. VPV was supported by CSIR fellowship and registered for PhD programme at Manipal University, Manipal. MK and AK are supported by UGC fellowship and registered for PhD programme at RCB, Faridabad.

## Conflict of interest

The authors declare that the research was conducted in the absence of any commercial or financial relationships that could be construed as a potential conflict of interest.

## Publisher’s note

All claims expressed in this article are solely those of the authors and do not necessarily represent those of their affiliated organizations, or those of the publisher, the editors and the reviewers. Any product that may be evaluated in this article, or claim that may be made by its manufacturer, is not guaranteed or endorsed by the publisher.
