# Supplementary material for: LigA formulated in AS04 or Montanide ISA720VG induced superior immune response compared to alum, which correlated to protective efficacy in a hamster model of leptospirosis
Source: Front Immunol. 2022 Oct 10;13:985802. doi: 10.3389/fimmu.2022.985802 (PMC9590693; doi:10.3389/fimmu.2022.985802)
Supplement: Supplementary file 1 [file DataSheet_1.pdf]

Supplementary Materials

Title: LigA formulated in AS04 or MontanideISA720VG induced superior immune response than Alum which correlated to protective efficacy in hamster model of Leptospirosis

Authors: Vivek P. Varma, Mohammad Kadivella, Ajay Kumar, Sridhar Kavela, and Syed M. Faisal*

*Correspondence to: faisal@niab.org.in or smfaisal77@gmail.com

The supplementary file contains-

Table 1- Survival data of immunized animals at 28-day post infection with virulent *Leptospira*

Table 2- Histopathology scores in various organs of the survived animals

Table 3- Primers used for RT-PCR

Table 1- Survival data of immunized animals at 28-day post infection with virulent *Leptospira*

**Group**	**Survival**		
	**Experiment 1**	**Experiment 2**	**Experiment 3**
**PBS**	0/6	0/6	0/6
**LAV-Alum**	4/6	2/6	3/6
**LAV-AS04**	5/6	3/6	4/6
**LAV-M**	5/6	4/6	5/6
**HKL**	6/6	5/6	6/6

Table 2- Histopathology scores in various organs of the survived animals

**Group**	**Pathology score**	
	Liver	Kidney
**PBS**	3,2,2,3,1,2	3,3,2,3,3,3
**LAV-Alum**	2,1,3,1,2,1	2,2,3,2,3,2
**LAV-AS04**	1,0,1,0,2,0	0,1,0,2,2,1
**LAV-M**	0,2,0,1,0,1	1,0,1,2,0,0
**HKL**	0,0,0,0,1,0	0,1,0,0,0,1

Table 3- Primers used for RT-PCR

Gene	**Primer Sequence**	
Beta actin	**F**	CACCCACACTGTGCCCATCTACGA
	**R**	GGATGCCACAGGATTCCATACCCA
**ccl2**	**F**	ACGTGTTGGCTCAGCCAGA
	**R**	ACTACAGCTTCCTTTGGGACACC
ccl3	**F**	ACTGCCTGCTGCTTCTCCTACA
	**R**	AGGAAAATGACACCTGGCTGG
**ccl5**	**F**	AGATCTCTGCAGCTGCCCTCA
	**R**	GGAGCACTTGCTGCTGGTGTAG
**ccl8**	**F**	CTTTGCCTGCTGCTCATAG
	**R**	GCACTGGATATTGTTGATTCTC
**ccl10**	**F**	TACTGCTGGCTCACCTC
	**R**	ATCTGTCTTGTGAAACCC
**ccl12**	**F**	GCTACCACCATCAGTCCTC
	**R**	CTGGCTGCTTGTGATTCTC
**ccr5**	**F**	ACACTCAGTATCATTTCTGG
	**R**	GGATCAGGCTCAAGATGACC
Il6	**F**	TGGAGTCACAGAAGGAGTGGCTAAG
	**R**	TCTGACCACAGTGAGGAATGTCCAC
**tnf-a**	**F**	ATAGCTCCCAGAAAAGCAAGC
	**R**	CACCCCGAAGTTCAGTAGACA
**ifn-g**	**F**	ACTCAAGTGGCATAGATGTGGAAG
	**R**	GACGCTTATGTTGTTGCTGATGG
**il-17**	**F**	TCCAGAAGGCCCTCAGACTA
	**R**	AGCATCTTCTCGACCCTGAA
**il-1b**	**F**	GCCTTGGGCCTCAAAGGAAAGAATC
	**R**	GGAAGACACAGATTCCATGGTGAAG
Mip1a	**F**	CCCAGCCAGGTGTCATTTTCC
	**R**	GCATTCAGTTCCAGGTCAGTG
**cxcl10**	**F**	CATGGTCCTGAGACAAAAGT
	**R**	TGATGACACAAGTTCTTCCA
**il10**	**F**	GCCAGAGCCACATGCTCCTA
	**R**	GATAAGGCTTGGCAACCCAAGTAA
**il5**	**F**	TGAGGCTTCCTGTCCCTACTCATAA
	**R**	TTGGAATAGCATTTCCACAGTACCC
cox2	**F**	TCTGGAACATTGTGAACAACATC
	**R**	AAGCTCCTTATTTCCCTTCACAC
tlr2	**F**	CTCCTGAAGCTGTTGCGTTAC
	**R**	GCTCCCTTACAGGCTGAGTTC
tlr4	**F**	TCGCCTTCTTAGCAGAAACAC
	**R**	GCCTTAGCCTCTTCTCCTTC
**foxp3**	**F**	GAGAGGCAGAGGACACTCAATG
	**R**	GCTCAGGTTGTGGCGGATG
**16s**	**F**	TAAAGGCTCACCAAGGCGAC
	**R**	TTAGCCGGTGCTTTAGGCAG
**Lipl32**	**F**	AAGCATTACCGCTTGTGGTG
	**R**	GAACTCCCATTTCAGCGATT
